# Intracellular Signaling by Diffusion: Can Waves of Hydrogen Peroxide Transmit Intracellular Information in Plant Cells?

**DOI:** 10.3389/fpls.2012.00295

**Published:** 2012-12-31

**Authors:** Christian Lyngby Vestergaard, Henrik Flyvbjerg, Ian Max Møller

**Affiliations:** ^1^Department of Micro- and Nanotechnology, Technical University of DenmarkKongens Lyngby, Denmark; ^2^Department of Molecular Biology and Genetics, Science and Technology, Aarhus UniversitySlagelse, Denmark

**Keywords:** diffusion, hydrogen peroxide, modeling, intracellular signaling, waves

## Abstract

Amplitude- and frequency-modulated waves of Ca^2+^ ions transmit information inside cells. Reactive Oxygen Species (ROS), specifically hydrogen peroxide, have been proposed to have a similar role in plant cells. We consider the feasibility of such an intracellular communication system in view of the physical and biochemical conditions in plant cells. As model system, we use a H_2_O_2_ signal originating at the plasma membrane (PM) and spreading through the cytosol. We consider two maximally simple types of signals, isolated pulses and harmonic oscillations. First we consider the basic limits on such signals as regards signal origin, frequency, amplitude, and distance. Then we establish the impact of ROS-removing enzymes on the ability of H_2_O_2_ to transmit signals. Finally, we consider to what extent cytoplasmic streaming distorts signals. This modeling allows us to predict the conditions under which diffusion-mediated signaling is possible. We show that purely diffusive transmission of intracellular information by H_2_O_2_ over a distance of 1 μm (typical distance between organelles, which may function as relay stations) is possible at frequencies well above 1 Hz, which is the highest frequency observed experimentally. This allows both frequency and amplitude modulation of the signal. Signaling over a distance of 10 μm (typical distance between the PM and the nucleus) may be possible, but requires high signal amplitudes or, equivalently, a very low detection threshold. Furthermore, at this longer distance a high rate of enzymatic degradation is required to make signaling at frequencies above 0.1 Hz possible. In either case, cytoplasmic streaming does not seriously disturb signals. We conclude that although purely diffusion-mediated signaling without relaying stations is theoretically possible, it is unlikely to work in practice, since it requires a much faster enzymatic degradation and a much lower cellular background concentration of H_2_O_2_ than observed experimentally.

## Introduction

It has long been clear that hydrogen peroxide is involved in signaling in plant cells, and a variety of mechanisms have been proposed for the information transmission (Neill et al., 2002). In a meta-analysis of gene expression induced by a range of localized stress in *Arabidopsis* leaves, Gadjev et al. (2006) showed that all, or almost all, localized stress treatments activated a large group of genes, which they named *general oxidative stress response markers*. However, each treatment regulated a unique group of genes, indicating that origin-specific signals were transmitted within the cell. This demonstrates a fundamental signaling phenomenon, which could be operating in any organs, in any cell type in response to any environmental condition.

We have proposed that oxidized peptides are better suited for transmitting specific information to the nucleus than hydrogen peroxide, the most likely signaling molecule in the Reactive Oxygen Species (ROS) family (Møller and Sweetlove, 2010). In that analysis, we concluded that the combination of cytoplasmic streaming and hydrogen peroxide degradation would make signaling via waves of hydrogen peroxide difficult, but the conclusion was not based on any mathematical modeling. More recently, Mittler et al. (2011) proposed that “the ROS signal itself carries within it a decoded (Note: the authors must have meant “encoded” here) message, much like calcium signals that have specific oscillation patterns within defined cellular locations. The specific features of the signal (amplitude, frequency, and/or localization) could then be perceived and decoded by specialized mechanisms to trigger specific gene expression patterns.”

We here consider to what extent intracellular hydrogen peroxide signaling via diffusion is possible, given the physical and biochemical conditions in the cytosol of plant cells. The proposed mechanism is similar to signaling in plant cells by calcium ions, where typically an external stimulus – an invading organism or a nearby cell – causes an influx of calcium ions across the plasma membrane (PM). These calcium ions diffuse through the cytosol to cause the release of calcium ions – either directly or indirectly via inositol triphosphate – from intracellular stores, typically the endoplasmic reticulum, which therefore acts as a relay station to maintain/amplify the signal. When release at the PM ceases, possibly via a negative feedback effect of the calcium ions, recovery occurs as calcium ions are pumped out of the cell or into intracellular stores. The calcium ion signal is received and decoded, e.g., by transcription factors, to change gene expression (Berridge, 1993; Parekh, 2011).

Here the cytoplasm (cytosol plus organelles) acts as an excitable medium, a non-linear dynamical system, and has the capacity to propagate a pulse, while it cannot support the passing of another pulse until it has had time to recover (known as the refractory time). The amplitude and propagation speed of the pulse is affected by the buffering capacity of the cytosol and molecular crowding (Jafri and Keizer, 1995; Dargan and Parker, 2003; Falcke, 2003; Mironova and Mironov, 2008).

When plant cells, e.g., detect an invading organism at the cell wall, one of a cell’s first responses is the recruitment of NADPH oxidase to the PM adjacent to the invasion site and the initiation of superoxide formation and ultimately hydrogen peroxide formation immediately outside the PM. The hydrogen peroxide can enter the cell via aquaporins in the PM (Bienert et al., 2007) and the cell *could* produce an amplitude- and/or frequency-modulated signal by synchronized modulation of the opening state of the aquaporin molecules in a membrane patch via phosphorylation/dephosphorylation (Maurel et al., 2009). We here address the *propagation* of such a signal, without which such a signal would make no sense.

We note that the presence of relaying stations in the cytosol is a prerequisite for calcium-mediated signaling in animal cells. We further note that no such relaying stations for H_2_O_2_ have been found in plant cells. We consequently consider how a diffusion-mediated signal propagates from the PM across the cytosol to the nucleus in a plant cell in the *absence* of relaying stations, and we investigate whether and when the signal can be delivered to the nucleus by diffusion alone.

We break the problem into its constituent parts and analyze it by starting with the simplest possible model, adding parts (and thus complexity) one at a time.

## Methods – Theory and Modeling

This section contains derivations of the theoretical description of diffusion-mediated signaling on which the results presented in Section “Results” are based. A list of the symbols used in the modeling is presented in Table 1.

**Table 1:** **List of symbols used in the mathematical modeling**.

Parameter	Explanation	Parameter	Explanation
*D*	Diffusion coefficient	Δ*t*	Time-interval during which the emitters (aquaporins) in the membrane stay open during the emission of a short pulse
▽^2^	Laplace operator, $\nabla^{2}=\frac{\partial^{2}}{\partial x^{2}}+\frac{\partial^{2}}{\partial y^{2}}+\frac{\partial^{2}}{\partial z^{2}}$	*t_D_*	Characteristic diffusion time
**r**	Vector describing the coordinates of a point in 3D space relative to the center of the source	*c*_max_	Peak concentration of the signal measured at the target
*x*	Distance from the source along the direction perpendicular to the cell membrane on which the source is located	*c*_trough_	Maximal concentration at which a consecutive signal can be detected after emission of an initial signal
*t*	Time after emission of a signal	α	Ratio between *c*_trough_ and *c*_max_. Defined here to be either 1/10 or 1/100
*G*	Fundamental solution to the diffusion equation	*t*_max_	Time after emission at which the peak concentration is achieved
*n_0_*	Amount of H_2_O_2_ molecules emitted by a single channel during a single pulse	*t*_ref_	Refraction time. Defined as the point in time when the concentration reaches *c*_trough_
ρ_*a*_	Density of emitters (aquaporins) in the area on the membrane which constitutes the source	*f*	Frequency of signal emission
*a*	Linear dimension of the source (area = *a* × *a*)	*V*_max_	Maximal enzymatic degradation rate
*L*	Length of the cell (in the closed geometry)	*K*_m_	Michaelis constant
erf	The error function, $erf\left(x\right)=\frac{2}{\sqrt{\Pi }}\int_{0}^{x}e^{-s^{2}}ds$	*v*	Flow speed in a cytoplasmic stream
erfc	The complementary error function, erfc(*x*) = 1 − erf(*x*)	*d*	Width of a cytoplasmic stream
*j*_0_	Amplitude of the flux over the membrane	Pe	Péclet number, which quantifies the relative importance of a flow compared to diffusion, Pe = *vd*/*D*

### The diffusion equation

The dynamics of the concentration *c* of a species of diffusing molecules is described by the diffusion equation

(1) $$\frac{\partial c}{\partial t}\left(\text{r},t\right)=D\nabla^{2}c\left(\text{r},t\right),$$

where **r** = (*x*, *y*, *z*) denotes the position in space, *t* parameterizes time, and ▽^2^ is the Laplace operator,

(2) $$\nabla^{2}=\frac{\partial^{2}}{\partial x^{2}}+\frac{\partial^{2}}{\partial y^{2}}+\frac{\partial^{2}}{\partial z^{2}}.$$

### Instantaneous point-like emitter, the fundamental solution

Consider a point-like emitter which emits an instantaneous pulse of *n*_0_ molecules at time *t* = 0. The concentration that is measured at a given distance $r=\sqrt{x^{2}+y^{2}+z^{2}}$ from the point of emission after a time *t* has elapsed, is then

(3) $$G\left(\text{r},t\right)=\frac{n_{0}}{{\left(4\pi Dt\right)}^{\frac{3}{2}}}e^{-\frac{r^{2}}{4Dt}},$$

which is the *fundamental solution* (http://en.wikipedia.org/wiki/Fundamental_solution, 2012-11-25) of the diffusion equation (Eq. 1).

### Emission from a single channel in a flat membrane

We next consider a single water channel in an infinite, flat impermeable membrane. We refer to this geometry as the *open geometry*. An aquaporin is less than 10 nm wide and is therefore effectively point-size compared to the distances over which the signal is transmitted (1–10 μm). We assume that the channel lets an instantaneous pulse of *n*_0_ molecules cross the membrane. The geometry is the same as above except for the membrane, which restricts the molecules to one half of space. The concentration in that half is hence twice that given in Eq. 3,

(4) $$c_{0}\left(\text{r},t\right)=\frac{2n_{0}}{{\left(4\pi Dt\right)}^{\frac{3}{2}}}e^{-\frac{r^{2}}{4Dt}}.$$

Equation 4 is the fundamental solution of the diffusion equation in the open geometry with its closed boundary condition at the membrane at *x* = 0,

(5) $$\frac{\partial c_{0}\left(\left(0,y,\;z\right),\;t\right)}{\partial x}=0.$$

### Synchronized emission from all channels in an area on the membrane

Now assume that channels are distributed evenly over the cell membrane. We denote by ρ*_a_* the surface density of these channels. If in an area of size *a* × *a* all of these emit simultaneously, then the emitted signal can be found by adding up the concentrations from all emitters in that area, using *c*_0_ for each emitter. That is, we find the concentration as the convolution of the fundamental solution (Eq. 4) with the constant density profile over the emission area.

(6) $$c\left(\text{r},t\right)=\int_{-\frac{a}{2}}^{\frac{a}{2}}\int_{-\frac{a}{2}}^{\frac{a}{2}}\frac{2n_{0}\rho_{a}}{{\left(4\pi Dt\right)}^{\frac{3}{2}}}e^{-\frac{x^{2}+{\left(y-y^{\prime }\right)}^{2}+{\left(z-z^{\prime }\right)}^{2}}{4Dt}dy^{\prime }dz^{\prime }.}$$

**Figure 1:**
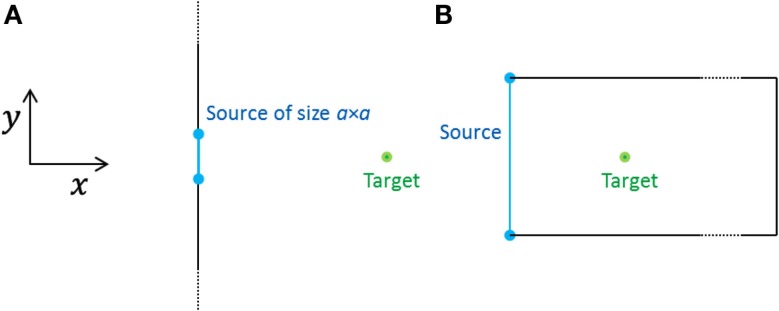
**Geometries of the two models considered, seen from above (the *z*-direction points out of the plane)**. **(A)** Open geometry: a square source emits into an infinite half-space. The target is situated at a distance *x* from the center of the source. **(B)** Closed geometry: the source fills the whole end-wall of a cell and the target is situated at a distance *x* from the center of the end-wall. All the cell walls are closed boundaries.

At a point on the *x*-axis a distance *x* from the membrane – i.e., on the normal through the center [(*y*, *z*) = (0, 0)] of the area containing channels (Figure 1A) – the measured concentration after emission is thus

(7) $$c\left(x,t\right)=\frac{N_{0}}{\sqrt{\pi Dt}}e^{-\frac{x^{2}}{4Dt}}\text{erf}{\left(\frac{a}{4\sqrt{Dt}}\right)}^{2},$$

where *N*_0_ = ρ*_a_n*_0_ and erf denotes the *error function*,

(8) $$\text{erf}\left(x\right)=\frac{2}{\sqrt{\pi }}\int_{0}^{x}e^{-s^{2}}ds.$$

Note that when we are far enough away from the source (approximately when *x* is larger than *a*), the signal looks the same no matter what the source looks like; the forms of signals from different emission areas are very similar, the only difference is the scale, which is determined by the total number of molecules emitted. The same similarity of signals occurs even close to the emission area at late times, when the initial shapes of all signals have disappeared by diffusion and all signals are described by Eq. 4 (albeit with different amplitudes). This also means that the specific shape of the emission area is not important. Signals emitted from a square, circular, or differently shaped areas all look alike as long as the dimensions of the areas are roughly the same.

### Open and closed geometries

For simplicity, we have assumed above that the dimensions of the cell are much larger than *a*, the linear extent of the area emitting the signal, i.e., the cell is effectively infinitely large (Figure 1A). We refer to this case as the *open geometry*, because most of the cell membrane is absent, effectively, being too far away to matter in the diffusive dynamics of concentrations of signaling molecules. In the Results section, Figures 3–7 present results for this geometry.

We shall see below that for the signal to be detectable at the target (∼10 μm from the source), the area of emission must be of the same order (∼10 μm × 10 μm) as the dimension of an epidermal cell (∼10 μm × 20 μm × 100 μm). In an extreme example, the whole end-wall (10 μm × 20 μm) of the cell emits a synchronized signal and the cell walls act as closed (reflecting) boundaries (Figure 1B). Because of the closed boundary conditions the concentration inside the cell does not depend on the *y*- and *z*- coordinates and this geometry is mathematically equivalent to a point source emitting in one dimension. Then the concentration measured at time *t* after emission only depends on the distance in the *x*-direction from the source and on the length *L* of the cell, e.g., *L* = 100 μm. It is calculated using the *method of images* (Griffiths, 1999) and is

(9) $$c\left(x,t\right)=\overset{\infty }{\underset{m=-\infty }{\sum }}\frac{N_{0}}{\sqrt{\pi Dt}}e^{-\frac{{\left(x-2mL\right)}^{2}}{4Dt}.}$$

In the Section “Results,” Figures 8–12 present results from such a closed system. Note that while the sum in Eq. 9 in principle is infinite, only a few terms with values of *m* near 0 contribute in practice.

### Refractory time

The refractory time *t*_ref_ is the time it takes, after detection of a pulse signal, for the concentration in a given point to drop to a sufficiently low value to allow detection of a new pulse signal. We define this threshold concentration to be some fraction α of the peak concentration *c*_max_ attained at that point in consequence of an emitted signal, the *trough/peak ratio*. This peak concentration occurs at *t*_max_ by definition of this time. In the simplest possible case of the point source, this time is equal to the diffusion time, defined as *t_D_* = *x*^2^/(6*D*) in three dimensions. In the simple case of an open geometry, one arrives at the same result, approximately, in cases where the target is further from the source than the size of the emission area. This approximation improves with distance from the source. In a closed geometry, *t*_max_ is approximately equal to *x*^2^/(2*D*), which is the diffusion time in one dimension. In general *t*_max_ must be found numerically.

The refractory time is found as the solution to the equation

(10) $$c\left(x,t_{ref}\right)=\alpha c\left(x,t_{\max}\right),$$

and is

(11) $$t_{ref}=-\frac{t_{D}}{W\left(-\frac{\alpha^{\frac{2}{3}}}{e}\right)}$$

in the simple cases of a point source as well as for an open geometry resembling a point source. Here *W* is the *Lambert-W function*, defined implicitly by *W*(*x*)*e^W^(*x*)* = *x*.

### Short pulse signal

An instantaneous pulse is a mathematical abstraction, which is easy to work with, but which does not exist in reality, since an instantaneous emission of a signal of some amplitude *N*_0_ would imply an infinite flux over the membrane. Instead, the channels stay open for a duration Δ*t* during which a flux *j*_0_ passes through, giving a total signal amplitude of *N*_0_ = *j*_0_Δ*t*. In the geometry described by Eq. 7, we cannot solve the diffusion equation analytically, and we must solve the diffusion equation (Eq. 1) numerically. This is done using *Comsol Multiphysics*, a finite element solver for physics and engineering applications.

However, for short times after emission, in the closed geometry or close to the source in the open geometry (i.e., in a point located at a distance from the source which is much smaller than the spatial extent of the source), the system is mathematically equivalent to that of a point source emitting in one dimension. Furthermore, at long times or far from the source in the open geometry, the system is equivalent to that of a point source emitter in three dimensions. Both these cases can be solved analytically. In the 1D case, for times *t* ≤ Δ*t*, the solution is

(12) $$c_{t\leq \Delta t}\left(x,t\right)=\frac{j_{0}}{D}\left[\sqrt{\frac{4Dt}{\pi }}e^{-\frac{x^{2}}{4Dt}}-x\;\text{erfc}\left(\frac{x}{\sqrt{4Dt}}\right)\right]$$

where erfc is the *complementary error function*, erfc = 1 − erf. For *t* > Δ*t*, the solution is

(13) $$c_{t>\Delta t}\left(x,t\right)=c_{t\leq \Delta t}\left(x,t\right)-c_{t\leq \Delta t}\left(x,t-\Delta t\right).$$

In the 3D case, the concentration measured at a distance *x* from the source along a line perpendicular to the membrane is for *t* ≤ Δ*t*,

(14) $$c_{t\leq \Delta t}\left(x,t\right)=\frac{j_{0}}{2\pi Dx}\text{erfc}\left(\frac{x}{\sqrt{4Dt}}\right),$$

and for *t* > Δ*t*,

(15) $$c_{t>\Delta t}\left(x,t\right)=c_{t\leq \Delta t}\left(x,t\right)-c_{t\leq \Delta t}\left(x,t-\Delta t\right).$$

Figure 3 illustrates these analytical results. As long as Δ*t* is approximately equal to or shorter than the characteristic diffusion time of an instantaneous pulse, the signal does not differ significantly from that of an instantaneous pulse. We refer to a signal of this type as a *short pulse signal*.

### Is the flux over the membrane constant?

As far as we know, H_2_O_2_ is mainly transported over the membrane by aquaporins. These are passive channels, so the concentration inside the membrane cannot exceed the concentration *c*_out_ outside the membrane. Furthermore, if the concentration inside the membrane approaches the outside concentration, the flux will diminish.

The maximal concentration on the inside of the membrane is found at time Δ*t* and is (from Eq. 12)

(16) $$c\left(0,\Delta t\right)=j_{0}\sqrt{\frac{4\Delta t}{D\pi }}.$$

This means that *j*_0_ must be much smaller than $\sqrt{\pi D∕4\Delta t}c_{\text{out}},$ or equivalently $\Delta t\ll \frac{\pi Dc_{\text{out}}^{2}}{4j_{0}^{2}}$ for the flux to stay constant during the time that the channels are open.

### Degradation of H_2_O_2_

Hydrogen peroxide is degraded by enzymes inside the cell. We assume Michaelis–Menten kinetics. Then the evolution of the concentration is described by the reaction-diffusion equation

(17) $$\frac{\partial c}{\partial t}\left(\text{r},t\right)=-\frac{V_{\max}c\left(\text{r},t\right)}{K_{\text{m}}+c\left(\text{r},t\right)}+D\nabla^{2}c\left(\text{r},t\right).$$

The reaction-diffusion equation is non-linear, and no known analytical solution exists. We solve the equation numerically using *Comsol*.

### Harmonically oscillating signal

We also consider a harmonically oscillating signal in the closed geometry, that is, a harmonically oscillating flux over the end membrane (*x* = 0) of the cell. This is described mathematically by the boundary condition

(18) $$\frac{\partial c}{\partial x}\left(0,t\right)=\frac{j_{0}}{D}\left(1-\cos\;2\pi ft\right),$$

where *j*_0_ is the average flux and *f* is the frequency of the signal. We solve the diffusion equation with degradation (Eq. 17) in one dimension numerically using *Comsol* with the boundary condition given by Eq. 18 at *x* = 0 and a closed boundary at *x* = 100 μm.

### Cytoplasmic streaming

**Figure 2:**
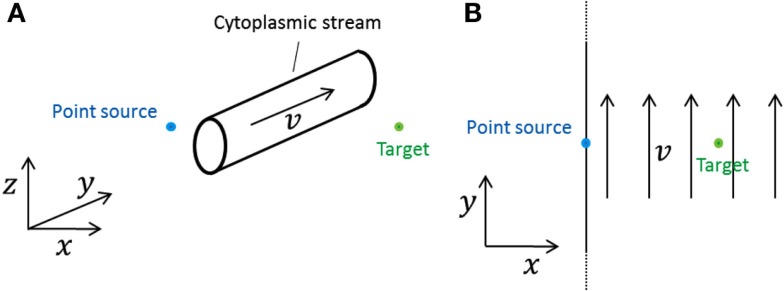
**Model systems considered for investigating the effect of cytoplasmic streams**. In both cases a point source emits into a cell which is of effectively infinite size (open geometry). **(A)** A roughly cylindrical cytoplasmic stream of effectively infinite extent along the *y*-axis and of diameter *d* is situated between the source and the target. The direction of the stream is perpendicular to the line between source and target. **(B)** A cytoplasmic stream fills up the entire cell. The direction of the stream is perpendicular to the line between source and target.

We investigate the effect that cytoplasmic streaming may have on signaling. A cytoplasmic stream can be modeled as a roughly cylindrical stream of infinite extent in the open geometry (Figure 2A). If a signaling molecule diffuses into this stream, it will be translated in the direction of the stream with a speed approximately equal to the average speed *v* found in a cross-section of the stream until the molecule diffuses out of the stream again. As Figure 2A shows, molecules can circumvent the stream and many will reach the target without having crossed the stream. Those whose do cross it will spend an amount of time in the stream which is on the order of the characteristic diffusion time,

(19) $$t_{D}=\frac{d^{2}}{4D}$$

where *d* is the width of the stream or the distance between the source and the target, whichever is smaller. During this time, a particle in the stream is translated a distance

(20) $$\Delta y=\frac{vd^{2}}{4D}=\frac{Pe}{4}d$$

in the direction of the stream (the *y*-direction). Here Pe is the *Péclet number*. In biological cells Pe is typically much smaller than one.

Since *d* is smaller than the distance from the source to the target, Δ*y* is negligible compared to the source-target distance if Pe is smaller than one. Thus, as long as Pe is smaller than one, the effect of cytoplasmic streams is negligible.

### Extreme example: An infinite cytoplasmic stream

In the previous subsection, we showed that cytoplasmic streaming has a negligible effect on signal transmission via diffusion in plant cells, by using an order-of-magnitude argument. The present subsection provides a more rigorous proof. It is rather equation-heavy and can be skipped.

We consider a cytoplasmic stream which fills the entire inside of the “cell” in the open geometry, moving with velocity *v* everywhere (Figure 2B). This is an extreme case; it is not physically realistic, but any realistic stream will do less transport of signaling molecules. So if we can show that this stream only has negligible effect on the signal measured at the target, then we know that a more realistic cytoplasmic stream, smaller and roughly cylindrical, e.g., has an even smaller effect.

At time *t* after emission of a short pulse signal from a point-like emitter located at (*x,y,z*) = (0,0,0), the concentration at the point **r** inside this cell is equal to

(21) $$c\left(\text{r},t\right)=\frac{2n_{0}}{{\left(4\pi Dt\right)}^{\frac{3}{2}}}e^{-\frac{x^{2}+{\left(y-vt\right)}^{2}+z^{2}}{4Dt}}.$$

The time *t*_max_ when the concentration reaches its peak at the point **r**, is found by setting the derivative of Eq. 21 to zero and solving for *t*. This gives

(22) $$t_{\max}=\frac{r}{v}\zeta ,$$

where

(23) $$\zeta =\sqrt{{\left(\frac{3}{Pe}\right)}^{2}+1}-\frac{3}{Pe}$$

and Pe is defined in Eq. 20. Measured at a distance *x* from the source and perpendicular to the stream (*y*, *z*) = (0, 0), the peak signal is then

(24) $$c_{\max}\left(x\right)=\frac{e^{-\frac{Pe\left(1+\zeta^{2}\right)}{4\zeta }}}{{\left(\frac{4\pi Dx\zeta }{v}\right)}^{\frac{3}{2}}}.$$

This should be compared to the peak signal in the absence of a cytoplasmic stream,

(25) $$c_{0,\max}\left(x\right)=\frac{e^{-\frac{3}{2}}}{{\left(\frac{2\pi r^{2}}{3}\right)}^{\frac{3}{2}}}.$$

Thus, the cytoplasmic stream decreases the peak concentration by a factor of

(26) $$\frac{c_{\max}\left(x\right)}{c_{0,\max}\left(x\right)}={\left(\frac{Pe}{6\zeta }\right)}^{\frac{3}{2}}e^{\frac{3}{2}-\frac{Pe\left(1+\zeta^{2}\right)}{4\zeta }}.$$

Even for a Péclet number of one, corresponding to a stream speed of 100 μm s^−1^ (the highest reported in literature) and a source-target distance of 10 μm (the longest considered), this factor is equal to 0.96. The cytoplasmic stream only decreases the peak signal by 4% in this extreme case. Furthermore, in this case the time at which the peak concentration is measured is 0.97 times *t_D_*, the time at which the peak concentration is measured in the absence of cytoplasmic streaming. So we can conclude that the effect of cytoplasmic streaming on the signal is completely negligible in more realistic, less extreme, scenarios.

### Typical biological parameter values

The values of the various parameters introduced above and the way they influence the hydrogen peroxide signal, will be treated stepwise in the Results section. Table 2 shows typical values of these parameters.

**Table 2:** **Parameters used in the modeling**.

Parameter	Interval studied	Comment and/or reference
Diffusion coefficient	*D* = 10^−9^ m^2^ s^−1^ = 10^3^ μm^2^ s^−1^	For diffusion in aqueous buffer *D* = 1.7 × 10^−9^ m^2^s^-1^ (van Stroe-Biezen et al., 1993); molecular crowding decreases *D* by approx. 20–50% (Straube and Ridgway, 2009)
Frequency	0.01–1 Hz	Assumed to lie in the same range as for Ca^2+^ (Jaffe, 1993)
Distance from source to target	1–10 μm	Typical cellular distances
Size of area on PM emitting synchronized pulse	Single channel – 10 μm × 10 μm	Relevant cellular dimensions
Assumed peak/trough ratio required to transmit signal	10–100-fold	
Minimal detection concentration	1–100 μM	Lower boundary is ten times the min. Ca^2+^ concentration measured in animal cells (Alberts et al., 1994); upper boundary is at the lower end of the average concentration measured in plant cells (Møller et al., 2007)
Maximal flux through a membrane channel	10^9^ molecules s^−1^ channel^−1^ = 1.7 × 10^−15^ mol s^−1^ channel^−1^	Total flux for both water and H_2_O_2_
Channel density	ρ*_a_* = 30 channel μm^−2^	Li et al. (2011)
Maximal H_2_O_2_ flux per μm^2^	10^−16^ mol μm^−2^s^−1^	Calculated using the maximal flux and the channel density and assuming no selectivity of the channels
Maximal degradation rate	*V*_max_ = 1–100 μM s^−1^	Lowest level estimated from Bonifacio et al. (2011)
Michaelis constant	20 μM for H_2_O_2_, values in the range 2–200 μM are considered	20 μM is the average for ascorbate peroxidases reviewed in Raven (2003)
Cytoplasmic streaming speed	1–100 μm s^−1^	Goldstein et al. (2008)
Cytoplasmic stream width	1–10 μm	Kristiansen et al. (2009)

## Results

Two properties of the emitted signal determine whether it can be detected at the target: (i) the *peak concentration*, *c*_max_, of the signaling molecules, which needs to be higher than the minimal detectable concentration, *c*_detect_, and (ii) the *refractory time* of the signal, which is the time one must wait between emission of signals for successive signals to be discernible. We defined this period as the time it takes for the concentration at the target to drop to say 1/10 (or, alternatively, to 1/100) of its maximal concentration. Exactly to what fraction of its peak that the signal must drop to before a new signal can be discerned is unknown and probably varies between cells. We therefore discuss results for both the relatively low and high arbitrarily chosen peak/trough ratios of 10 and 100.

If we assume that the resting concentration of H_2_O_2_ in the cytosol is 0.1 μM (by analogy with free calcium ions in cells) and that the peak concentration must at least be ten times higher than this to be detected, then the minimal detectable concentration at the target is 1 μM for a peak/trough ratio of 10 and 10 μM for a peak/trough ratio of 100.

### Maximal flux over the cell membrane

H_2_O_2_ crosses the PM through aquaporins. The maximal flux through a single aquaporin channel is on the order of 10^9^ molecules channel^−1^ s^−1^ (Jensen and Mouritsen, 2006) while the number of channels per μm^2^ is on the order of 30 (Li et al., 2011). This gives a maximal flux per μm^2^ of 3 × 10^10^ molecules μm^−2^ s^−1^, or in units of moles: 5 × 10^−14^ mol μm^−2^s^−1^. If we assume that the aquaporins allow H_2_O_2_ and water molecules to pass with rates proportional to their respective concentrations and we further assume that the concentrations outside the PM are 100 mM for H_2_O_2_ (very high) and 55 M for water (standard), then the maximal flux of H_2_O_2_ over the cell membrane is 10^−16^ mol μm^−2^ s^−1^.

A prerequisite for attaining this maximal flux is that the concentration of H_2_O_2_ outside the membrane is much higher than the concentration inside, since the aquaporins are passive channels. This is, however, always the case in practice, since even if we let the channel stay completely open for 1 s, the concentration will maximally reach 4 mM inside the cell.

### Short pulse signal

A signal is emitted at the PM by opening the aquaporins for a while. We first consider signals consisting of short pulses. All other types of signals can be constructed by adding short pulses and they are the type of signal, which allows the fastest frequency modulation. A short pulse is obtained by fast opening and closing of the channels in the membrane. For mathematical convenience this can be idealized to an instantaneous pulse where the channels are opened and closed infinitely fast and the flux over the membrane is infinite during this infinitesimal time-interval.

**Figure 3:**
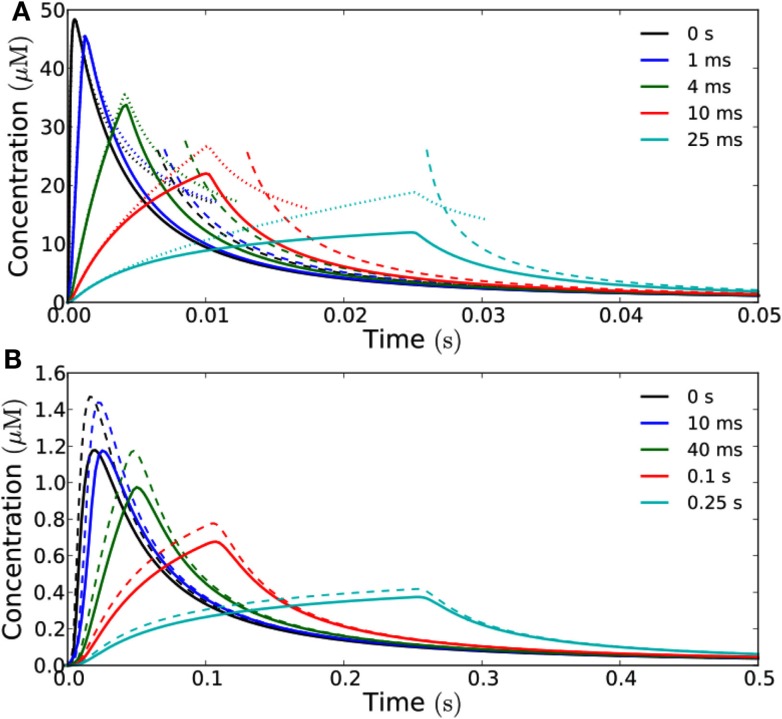
**How opening time of the channels influence the signal received at the target**. Concentration as a function of time measured at a distance of 1 μm **(A)** and 10 μm **(B)** from a square emission patch of size 10 μm × 10 μm in the open geometry. The curves have been rescaled such that they correspond to the same total signal, i.e., the flux is adjusted such that the total amount of H_2_O_2_ emitted is the same for the different opening times and equal to 10^−19^ mol μm^−2^. The time-interval in which the channels stay open is varied between 0 and 25 ms **(A)** and 0 and 0.25 s **(B)**. Solid curves represent exact results for a 10 μm × 10 μm area obtained from Comsol simulations while dashed lines represent analytical results for a point source. **(A)** Dotted lines represent the analytical (1D) solution for an infinite source area. Note how the exact results agree well with the analytical solutions for an infinite source for short times, since we are so close to the source that it effectively feels infinite at this short time-scale. At long times the analytical solutions corresponding to emission from a point source agree well with the exact results and all the curves collapse on the same curve. The diffusion time *t_D_* of a signal emitted in an instantaneous pulse is equal to 1 ms. **(B)** At a distance of 10 μm from the source, the exact results are well approximated by the analytical solution for a point source emitter. The peak signal from a point source is slightly higher than from the 10 μm × 10 μm area since the initial point source signal is more concentrated. However, the curves are qualitatively similar and they all collapse to the same curve at long times as in **(A)**. The diffusion time *t_D_* for an instantaneous pulse signal is here equal to 25 ms.

Increasing the time the channels stay open naturally increases the signal amplitude. At long times after emission, there is a simple proportionality between the time the channels stay open (and thus the total signal) and the concentration measured at the target; as with the different emission areas the curves describing the concentration all collapse to the same on curve (Figure 3), except for a scaling factor determined by the total amount of H_2_O_2_ emitted. That is, at sufficiently long times the curves become equal except for a scaling factor.

For times shortly after the emission of the signal, it is another story. Longer opening time flattens out the peak of the concentration, and the peak concentration, relative to the concentration measured at long times, is decreased. This in turn increases the refractory time.

When we consider a target that is 1 μm away, the signal can be considered a short pulse as long as the time during which the aquaporins stay open is approximately 1 ms or shorter, since its relative amplitude does not differ significantly from that of an instantaneous pulse (Figure 3A). The maximal amount of H_2_O_2_ that can be released in a single short pulse per unit area, i.e., the maximal amplitude of a short pulse signal, is then 10^−19^ mol μm^−2^. At a distance of 10 μm, the signal can be considered a short pulse, if the aquaporins stay open for approximately 10 ms or less (Figure 3B), i.e., the maximal amplitude of such a short pulse is 10^−18^ mol μm^−2^. Here, where the source-target distance (10 μm) is comparable to the dimensions of the source (10 μm × 10 μm), we also see that the signal received at the target is well approximated by the analytical solution for a point source (Figure 3B).

### The size of the emission area

The size of the activated area on the PM, the area creating the synchronized signal, may vary depending on, e.g., what caused the signal. This gives different signals. We consider emission area sizes ranging from a single channel to 100 μm^2^: (i) a single channel, (ii) 1 μm × 1 μm (a small patch), and (iii) 10 μm × 10 μm (a large patch extending over a major fraction of one side of a plant cell).

**Figure 4:**
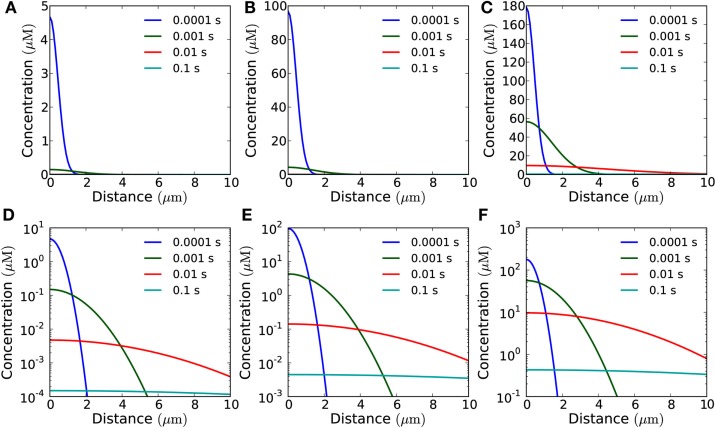
**Concentration of H_2_O_2_ as a function of distance from the membrane in the open geometry**. Snapshots of the concentration profile at different points in time after emission of a single pulse of 10^−19^ mol μm^−2^ from areas of varying size: **(A)** for a single channel; **(B)** for an area of 1 μm × 1 μm (containing 30 channels); **(C)** for an area of 10 μm × 10 μm (3,000 channels). **(D–F)** Same plots as in **(A–C)** shown with logarithmic *y*-axis.

**Figure 5:**
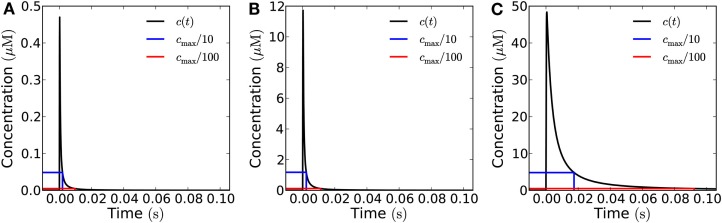
**Influence of the emission area on the measured signal at a target 1 μm from the source in the open geometry**. Concentration of H_2_O_2_ as a function of time at a distance of 1 μm from the source after emission of a single pulse of 10^−19^ mol μm^−2^ from areas of varying size. The blue (red) lines mark 1/10th (1/100th) of the peak concentration and the time at which it is obtained. **(A)** for a single channel (signal: 3 × 10^−21^ mol) the refractory times are 1.9 and 9.6 ms depending on whether the peak/trough ratio is 10 or 100, respectively. **(B)** For a 1 μm × 1 μm area (30 channels, total signal: 10^−19^ mol), refractory times: 2.3 and 11 ms. **(C)** For a 10 μm × 10 μm area (3000 channels, total signal: 10^−17^ mol), refractory times: 19 and 100 ms. Note that the signal from a 1 μm × 1 μm area **(B)** and a point emitter **(A)** are almost identical when measured at a distance of 1 μm. This is also reflected in the refraction times.

**Figure 6:**
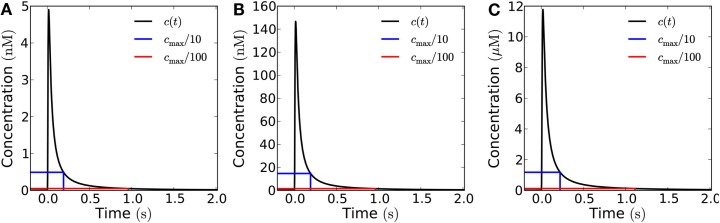
**Influence of the emission area on the measured signal at a target 10 μm from the source**. Concentration of H_2_O_2_ as a function of time at a distance of 10 μm from the source after emission of a single instantaneous pulse of 10^−18^ mol μm^−2^ H_2_O_2_ from areas of varying size in the open geometry. The blue (red) lines mark 1/10th (1/100th) of the peak concentration and the time at which it is obtained. **(A)** For a single channel (signal: 3 × 10^−20^ mol) the refractory times are 0.19 and 0.96 s depending on whether the peak/trough ratio is 10 or 100, respectively. **(B)** For a 1 μm × 1 μm area (total signal: 10^−18^ mol), refractory times: 0.19 and 0.96 s. **(C)** For a 10 μm × 10 μm area (total signal: 10^−16^ mol), refractory times: 0.22 and 1.1 s. The time after signal emission where the concentration has dropped to 1/10 and 1/100 of its maximal value, are marked by vertical lines. All the three curves look almost identical except for the scale factor, i.e., the signals in **(B,C)** are practically indistinguishable from that of a point source **(A)**. Comparison of the refraction times in the three cases also shows this.

Varying the area of the patch from where molecules are emitted, affects the total amplitude of the signal, if other parameters are kept constant (Figure 4). Furthermore, a larger emission area means slower decay, i.e., longer refractory time (Figures 5 and 6).

For a signal emitted by a single channel, it is clear that we would need a very high initial signal amplitude to be able to detect the signal, even as close as 1 μm away. If we assume that the resting concentration of H_2_O_2_ is 0.1 μM and that we can detect the signal at a concentration 10-fold higher than this, we need a signal twenty times larger than what a single channel can emit in a short pulse, to be able to detect the signal from a single channel at 1 μm from the source. Thus, in the following we will only consider emission from sources of size 1 μm × 1 μm and 10 μm × 10 μm.

### Distance from the source

The refractory time does not depend on the signal amplitude, but depends highly on distance from the source and emission patch size (Figures 5 and 6). Increasing the distance between the source and the target increases the refractory time and, additionally, drastically decreases the amplitude of the signal (Figures 5 and 6). In the open geometry the main obstacle to signaling is the difficulty of attaining a peak concentration at the target which is sufficiently high to be detected.

At a distance of 1 μm (Figure 5), emission of a pulse of 10^−19^ mol μm^−2^ from an area of 1 μm × 1 μm (total signal is 10^−19^ mol) creates a detectable signal with a refractory time of 2.3 ms for a peak/trough ratio of 10 (11 ms for a ratio of 100), indicating that in the open geometry frequencies of up to 500 Hz are feasible even without enzymatic degradation of H_2_O_2_. When the area is larger, 10 μm × 10 μm (total signal: 10^−17 ^mol), the signal is considerably stronger, but the refractory time increases to 19 ms (100 ms for a peak/trough ratio of 100), indicating that here frequencies up to 50 Hz are possible.

With distance, the wave broadens, thereby increasing the refractory time. At a distance of 10 μm (Figure 6), only an emission from the largest area considered (10 μm × 10 μm) is detectable, even if the detection limit is as low as 1 μM. Here the refractory time is 0.22 s for a peak/trough ratio of 10 (1.1 s for a ratio of 100), indicating that signaling with frequencies up to 5 Hz is feasible.

In general, as long as the distance between the target and the source is greater than or equal to the dimensions of the area of emission, the signal is well approximated by the signal from a point source (compare Figures 5A,B and 6A–C) and the refractory times are given approximately by the analytical result for a point emitter (Eq. 11). In this case, for a source-target distance of 1 μm, the refractory times for the signal to reach 1/10th and 1/100th of its peak value for a point source are 1.9 and 9.6 ms respectively. For a source-target distance of 10 μm, the refractory times for a point source are 0.19 and 0.96 s.

### Signaling with trains of short pulses

**Figure 7:**
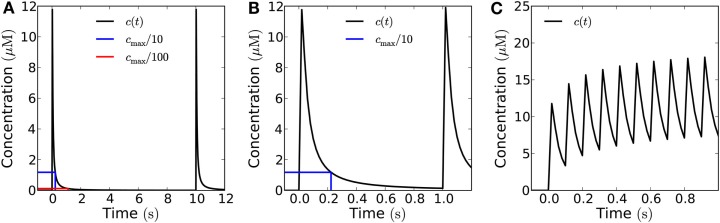
**Recorded signal at a distance of 10 μm from emitter of a train of pulses in the open geometry**. The individual pulse amplitude is 10^−18^ mol μm^−2^ emitted from a 10 μm × 10 μm area (total individual signal: 10^−16^ mol). The blue (red) lines mark 1/10th (1/100th) of the peak concentration and the time at which it is obtained. **(A)** For an emission frequency of 0.1 Hz both a 10-fold and a 100-fold decrease of the signal is easily detected between emission of signals. **(B)** For an emission frequency of 1 Hz, a 100-fold difference is no longer obtained, the concentration only has time to decrease by a factor of 85 before the arrival of a new signal. **(C)** For an emission frequency of 10 Hz, only a maximal peak/bottom ratio of around two is seen at the target.

Figure 7 shows what a train of short pulses emitted from a 10 μm × 10 μm area looks like at a distance of 10 μm from the source. At a low frequency, say 0.1 Hz, the H_2_O_2_ concentration has time to return to the resting level (Figure 7A), at 1 Hz, the level does not quite return to 1/100th of the peak value since the refractory time is 1.1 ms (Figure 7B), and at a frequency of 10 Hz there is a build-up of H_2_O_2_ and only a twofold difference between peak and trough, insufficient for the signal to be deciphered (Figure 7C).

### Finite cell size and removal of H_2_O_2_

We have until now assumed that the cell is so large that there is no effect of cell walls on the distribution of H_2_O_2_, except from the wall through which it enters the cell. In reality, and especially when we look at repeated pulses and other cases of continuous signaling, H_2_O_2_ will build-up in the cytosol and thereby prevent further signaling, unless it is removed.

We consider a closed geometry in which the signal is emitted from the whole end-wall of the cell, e.g., an area of 10 μm × 20 μm. This is the most restricted geometry that one can imagine, i.e., it complements the open geometry treated above and thereby delimits the range of possible phenomena. This is the geometry in which one can have the highest signal amplitudes, but on the other hand also the longest refractory times.

**Figure 8:**
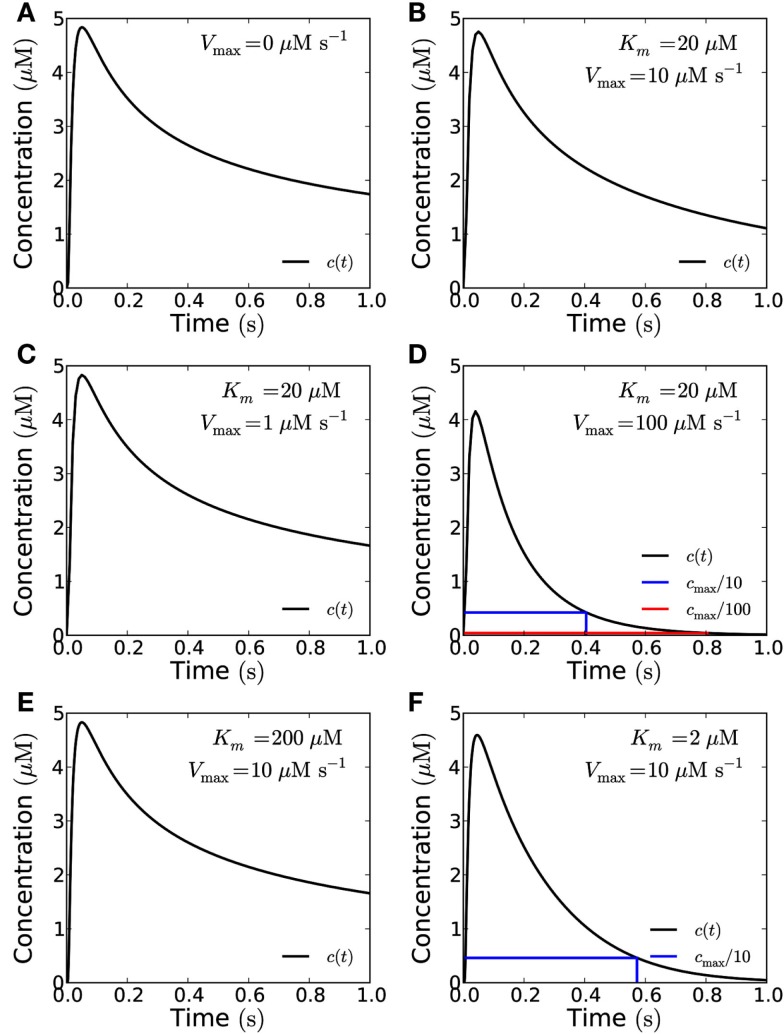
**The effect of enzymatic degradation of H_2_O_2_ on a short pulse signal**. The signal is recorded at a distance of 10 μm from the source in the closed geometry. Enzymatic degradation of H_2_O_2_ reduces the refractory time, but does not significantly reduce the peak concentration. A signal of amplitude 10^−19^ mol μm^−2^ is emitted from the whole end-wall (10 μm × 20 μm area, total signal: 2 × 10^−17^ mol) of a 100 μm long cell and measured at a distance of 10 μm. H_2_O_2_ is reflected by the sides of the cell. **(A)** No degradation. **(B)** For degradation with *V*_max_ = 10 μM s^−1^ and *K*_m_ = 20 μM. **(C)** *V*_max_ = 1 μM s^−1^ and *K*_m_ = 20 μM. **(D)** *V*_max_ = 100 μM s^−1^ and *K*_m_ = 20 μM. Here the refraction times are 0.4 s and 0.8 for 1/10th (blue lines) and 1/100th (red lines) of the peak concentration *c*_max_. **(E)** For *K*_m_ = 200 μM and *V*_max_ = 10 μM s^−1^. **(F)** *K*_m_ = 2 μM and *V*_max_ = 10 μM s^−1^. Here the refraction time to reach 1/10th of the peak concentration is 0.57 s. The effect of lowering (raising) *K*_m_ is qualitatively equivalent to the effect of raising (lowering) *V*_max_.

**Figure 9:**
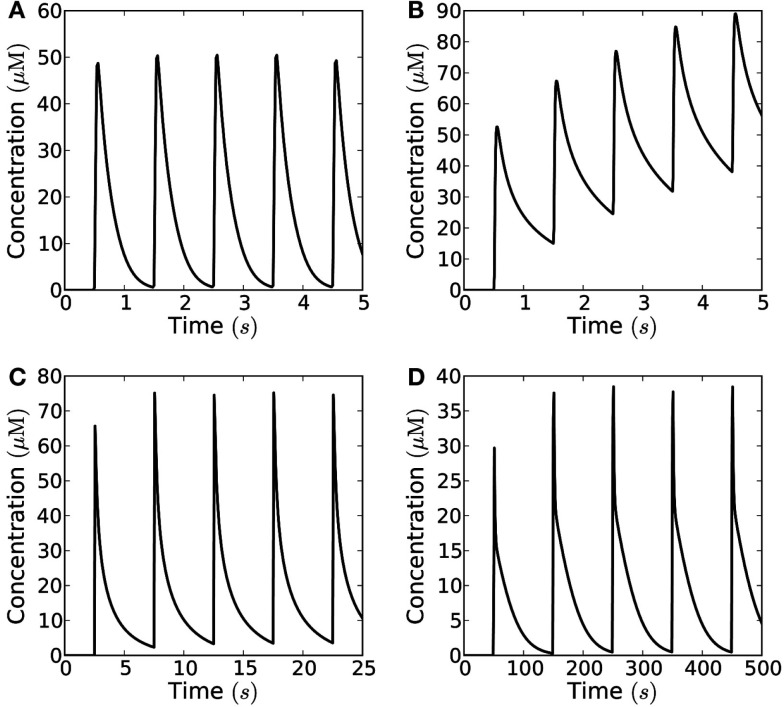
**Frequency-modulated signals consisting of short pulses**. The range of frequencies at which signaling is possible in the closed geometry, depends strongly on the maximal degradation rate of H_2_O_2_. Concentration measured at 10 μm distance from an area of 10 μm × 10 μm, which emits short pulses; the signaling molecules are enzymatically degraded in a reaction with *K*_m_ = 20 μM and *V*_max_ = 1−100 μM s^−1^, the amount of H_2_O_2_ released by each pulse is *N*_0_ = 10^−18^ mol μm^−2^. **(A)** For fast degradation (*V*_max_ = 100 μM s^−1^) high frequency (*f* = 1 Hz) and high amplitude signaling is possible; **(B)** for an intermediate degradation rate (*V*_max_ = 10 μM s^−1^) signaling is not possible at the highest frequencies (i.e., *f* = 1 Hz), since the signal’s refractory time is too long; **(C)** at frequencies lower than 0.2 Hz signaling is possible for *V*_max_ = 10 μM s^−1^; **(D)** For slow degradation (*V*_max_ = 1 μM s^−1^) only low frequency signaling is possible (*f* = 0.01 Hz).

If no H_2_O_2_ is removed or degraded, signaling over 10 μm is not possible since the cell fills up with H_2_O_2_ and the concentration settles at a constant value throughout the cell of approximately 20% of $c_{\text{peak}}$ as measured at 10 μm from the source. However, there are several mechanisms by which H_2_O_2_ can be removed – transport out of the cell or into organelles, chemical reactions, and enzymatic reactions. We cannot evaluate the magnitude of the former two and if transport out of the cell is passive it will counter the effect of the cell walls to some extent, i.e., the system will be described by the open geometry or some intermediary between the closed and open geometries. So here we will let H_2_O_2_ removal consist entirely of enzymatic degradation to analyze how H_2_O_2_ removal affects signaling. The cytosol contains several enzymes capable of removing hydrogen peroxide, e.g., ascorbate peroxidase. When we include removal of H_2_O_2_ by Michaelis–Menten kinetics in the model, we see that signaling over 10 μm becomes possible at frequencies up to 2.5 Hz (for a peak/trough ratio of 10) when the removal rate is 100 μM s^−1^ (Figures 8D and 9A). At a much lower removal rate (1 μM s^−1^) only signaling at rates up to 0.06 Hz is possible (for a peak/trough ratio of 10, 0.01 Hz for a ratio of 100, Figure 8C). Since the diffusion of H_2_O_2_ is fast on intracellular length scales, degradation does not affect the maximal concentration of H_2_O_2_ much (Figure 8), though it significantly decreases the refractory time. At an intermediate removal rate (10 μM s^−1^), similar to the one reported by Bonifacio et al. (2011) signaling at 0.2 Hz is possible (Figures 9B,C). In all cases peak concentrations of 50 μM at the target can be reached.

The Michaelis constant *K_m_* may change as well, i.e., due to molecular crowding (see Effect of Molecular Crowding). We have considered a ten-fold increase or decrease in *K_m_* (i.e., to 200 or 2 μM) and see that the effect of an increase in *K_m_* is qualitatively the same as the effect of a corresponding decrease of *V*_max_ and *vice versa* (Figures 8E,F).

Frequencies, amplitudes, and background H_2_O_2_ concentrations, which make signaling possible are listed in Tables 3 and 4.

**Table 3:** **Possible parameter values for signaling over a distance of 1 μm using short pulses (open geometry)**.

Parameter	Approx. max. value	Comments
Frequency	500 Hz	For *c*_max_/*c*_trough_ = 10
	90 Hz	For *c*_max_/*c*_trough_ = 100
Amplitude	10–50 μM	For a 1 ms pulse and depending on the size of the emission area (1–100 μm^2^)
	20–250 μM	For a 10 ms pulse; here the max. frequency is decreased by a factor two
Maximum background concentration	5–25 μM	For a 10 ms pulse and *c*_max_/*c*_trough_ = 10

**Table 4:** **Possible parameter values for signaling over a distance of 10 μm using short pulses (closed geometry)**.

Parameter	Approx. max. value	Comments
Frequency	1–2 Hz	For *V*_max_ = 100 μM s^−1^, depending on *c*_max_/*c*_trough_
	0.2 Hz	For *V*_max_ = 10 μM s^−1^
	0.01 Hz	For *V*_max_ = 1 μM s^−1^
Amplitude	50 μM	For 10 ms pulses
	500 μM	For 0.1 s pulses, only possible for *V*_max_ = 100 μM s^−1^, and a max. frequency of 0.5 Hz
Maximum background concentration	5 μM	For 10 ms pulses and *c*_max_/*c*_trough_ = 10

### Harmonically oscillating signal

Thanks to the late French physicist Joseph Fourier (the inventor of Fourier analysis), we know that any signal can be constructed as a sum of harmonic functions, i.e., sines and cosines. This type of signal is complimentary to the short pulse, since it is regulated gradually, whereas the short pulse is regulated by an abrupt open/close mechanism. The manner in which such a signal propagates thus gives us a new angle on the problem of signal transmission. This angle is the natural one to use in an investigation of the propagation of pulse trains, because a harmonic signal is the simplest possible periodic pulse train as seen from a mathematical point of view, and because more complicated pulse trains can be viewed as super-positions of these simple ones for analytical purposes. That is the essence of Fourier analysis. Add to this that harmonic signals are the carrier waves for signals encoded by frequency modulation.

While the non-linear kinetics of the Michaelis–Menten degradation term prevents us from solving the kinetic equation using Fourier analysis, that analysis still applies to the emitted signal. It is the interactions of different Fourier components of the propagating signal in the degradation term that bars Fourier analysis.

**Figure 10:**
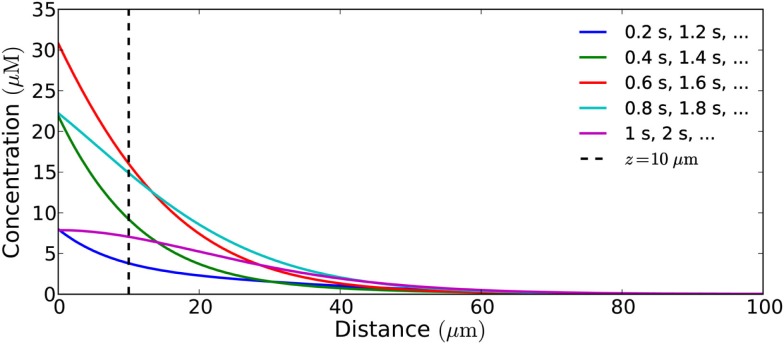
**Snapshots of the concentration profile in a cell of length 100 μm (closed geometry) of a signal from a harmonically (sinusoidal) emitting source, taken at different times in the emission cycle**. No wave-like pattern is seen. This is a signature feature of signal propagation by diffusion. The wave is only in the time-domain and can be seen if one continuously monitors the concentration of H_2_O_2_ at a given distance, e.g., at 10 μm from the source. The period of the flux is 1 Hz, its amplitude is 10^−18^ mol μm^−2^s^−1^, and *V*_max_ is 100 μM s^−1^.

**Figure 11:**
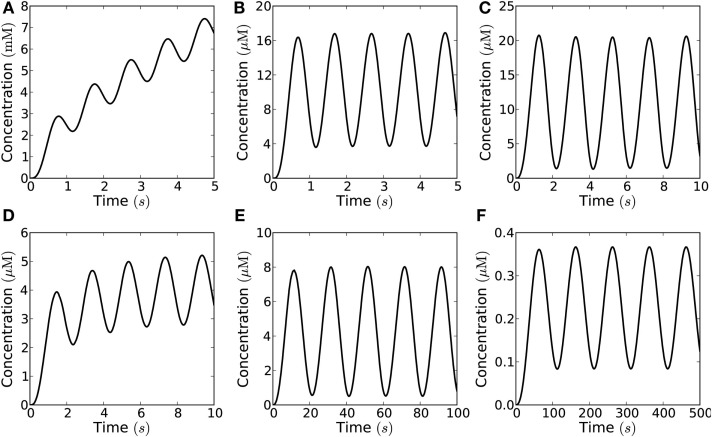
**Harmonically oscillating signals in theory allows much higher signaling amplitude than short pulses**. However, in practice the amplitude needs to be limited to allow signaling at relevant frequencies, thus harmonic signals actually allow a lower signaling amplitude than short pulses at similar frequencies. Concentration measured at a distance of 10 μm from the emission area of 10 μm × 10 μm in the closed geometry, which emits a harmonic (sinusoidal) flux with amplitude *j*_0_ and frequency *f* = 1−0.01 Hz; the signaling molecules are enzymatically degraded in a reaction with *K*_m_ = 20 μM and *V*_max_ = 1−100 μM s^−1^. **(A)** Even for maximal degradation rate (*V*_max_ = 100 μM s^−1^) the maximal flux of 10^−16^ mol μm^−2^s^−1^ is so high that the concentration of molecules build-up over time and no signaling is possible; **(B)** for a lower flux of 10^−18^ mol μm^−2^s^−1^ the concentration no longer builds up over time, but fast signaling is still not possible (*f* = 1 Hz) due to the long refractory time; **(C)** for a lower frequency (*f* = 0.5 Hz) signaling is possible (with *c*_max_/*c*_trough_ = 10); **(D)** if the degradation rate is lower (*V*_max_ = 10 μM s^−1^) signaling at 0.5 Hz is no longer possible; **(E)** the frequency must be lowered accordingly to allow signaling (*f* = 0.05 Hz); **(F)** for a degradation rate of 1 μM s^−1^ no signaling is possible, even with the lowest relevant frequencies (*f* = 0.01 Hz) and at very low amplitude (*j*_0_ = 10^−21^ mol μm^−2^s^−1^).

Figure 10 shows what a signal with a harmonic source looks like throughout the cell at different points in the emission cycle, i.e., snapshots of the concentration profile at different points in time. No spatial wave-like pattern is seen. This is a signature feature of diffusive transport in a non-excitable medium. This is because signal propagation is only driven by concentration gradients and the concentration of signaling molecules thus always will be highest at the source. The wave-pattern is only seen in the time-domain, e.g., when we follow the concentration in a single point over time (Figure 11). To see spatial wave-patterns in diffusion-mediated signaling a non-linear, excitable medium is required.

**Figure 12:**
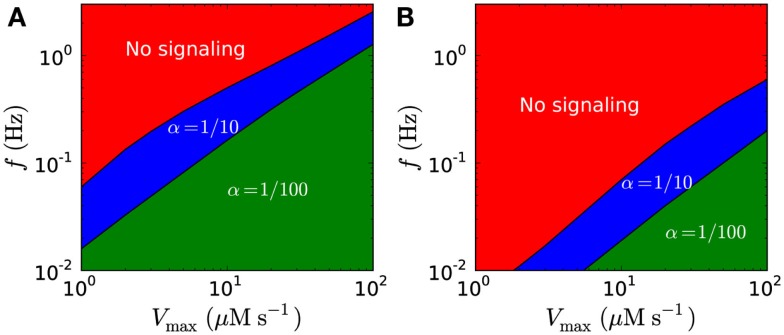
**Possible signaling frequencies *f* in the closed geometry as a function of *V*_max_**. Shown for trough/peak ratios of α = 1/10 (blue) and α = 1/100 (green). **(A)** Possible frequencies for short pulse signaling. **(B)** Possible frequencies for harmonic signaling. Higher frequencies are obtainable for short pulse signaling than for harmonic signaling. In both **(A,B)**, the signal detection threshold is 1 μM for α = 1/10 and 10 μM for α = 1/100, and *K*_m_ is 20 μM.

Though harmonic signals in theory allow much higher signaling amplitudes, they are limited by the refractory time, and the possible peak concentrations are lower for harmonic signals than for short pulse at similar frequencies (Figure 11; Table 5). Figures 12A,B shows possible signaling frequencies as a function of *V*_max_ for signaling using short pulses (Figure 12A) and a harmonically oscillating flux (Figure 12B).

**Table 5:** **Possible parameter values for signaling over a distance of 10 μm using harmonic signals (closed geometry)**.

Parameter	Approx. max. value	Comments
Frequency	0.5 Hz	For *V*_max_ = 100 μM s^−1^
	0.05 Hz	For *V*_max_ = 10 μM s^−1^
	<0.01 Hz	For *V*_max_ = 1 μM s^−1^
Amplitude	10–20 μM	For *V*_max_ = 100 μM s^−1^ and *f* = 0.5 Hz. Can be higher for lower frequencies; lower *V*_max_ implies lower amplitude
Maximum background concentration	1–2 μM	For *c*_max_/*c*_trough_ = 10 and depending on *V*_max_

### Effect of molecular crowding

Molecular crowding effectively lowers the diffusion coefficient of a molecular species in the cytosol compared to its value in water. For calcium ions in animal cells, this effect is of the order of 20–50% (Straube and Ridgway, 2009). We have assumed a similar effect on the diffusion coefficient of H_2_O_2_ in plant cells, lowering the diffusion coefficient of H_2_O_2_ from 1,700 μm^2^ s^−1^ (van Stroe-Biezen et al., 1993) to 1,000 μm^2^ s^−1^. Excluding the effect of degradation, a twofold increase of the diffusion coefficient will simply decrease the characteristic diffusion time and refractory times by a factor two, but will not influence signal amplitudes; an increase of the diffusion coefficient diminishes the influence of degradation, since it reduces the time-scale of signaling.

Molecular crowding may also affect the binding rates and rates of catalysis of enzymes and thus change *K_m_* and *V*_max_ (Zhou et al., 2008). A change of the rate of catalysis changes both *K_m_* and *V*_max_ in the same direction, which means that these two effects largely cancel each other in this case (Figure 8). A change in the association rate between signaling molecules and the degradation enzymes only affects *K_m_* and will thus to a larger extent affect reaction kinetics. The effect of crowding varies highly for different enzymes (Norris and Malys, 2011) and is unknown for enzymes degrading H_2_O_2_ in plant cells. So we have considered a wide range of possible values for *K_m_* (2–200 μM, Figure 8).

### Effect of cytoplasmic streaming

Finally, we have investigated what effect cytoplasmic streaming may have on signaling. We have considered a roughly cylindrical cytoplasmic stream in between the source and the target (Figure 2A). A simple order-of-magnitude argument showed that the influence of the stream is negligible if the Péclet number is on the order of one or smaller. Here the Péclet number is defined as the ratio between the stream’s width times the stream velocity and the diffusion coefficient. Considering the maximal stream speed reported in the literature, *v* = 100 μm (Goldstein et al., 2008; Table 2), and considering a stream which is as wide as the largest distance between the source and the target, 10 μm, we get a Péclet number of one. This means that even in this extreme case, cytoplasmic streaming does not significantly disturb signaling.

Thus, cytoplasmic streaming does not hinder intracellular signaling by diffusion, since the diffusion coefficient is so large compared to typical values of the cytoplasmic stream width and speed (Table 2). On the contrary, it lowers the refractory time, because the stream has a stronger delaying effect on the signal molecules that arrive late at the target than it has on the early arrivers, due to the former spending more time in the stream than the latter. The net effect is a sharper profile of concentration plotted against time, similar to the situation in which signal molecules are degraded.

## Discussion

Our modeling shows that diffusion-mediated frequency- and amplitude-modulated signaling with H_2_O_2_ over distances of up to 10 μm is possible if we assume that the detection limit is low (on the order of 1 μM), the synchronized emission area is large (Figures 6 and 8), the flux over the membrane is high, and the removal rate is high (Figures 8 and 9). Under these conditions, signaling with a frequency of up to 2 Hz at 10 μm is possible with amplitudes up to 50 μM (Figures 8, 9, and 12A; Table 4).

The effect of enzymatic degradation of H_2_O_2_ is the opposite of what was proposed by Møller and Sweetlove (2010). Instead of making signaling difficult, a high rate of H_2_O_2_ degradation is actually a prerequisite for signaling as it reduces the refractory time (Figures 7–9 and 11). Other possible removal mechanisms are uptake into organelles and/or transport out of the cell, neither of which has been described for plant cells and both of which have the same overall effect as degradation, in as much as they would reduce the refractory time. The rate of H_2_O_2_ removal is a parameter that the plant cell may modulate to accommodate a wider range of signaling frequencies.

Also contrary to what was proposed by Møller and Sweetlove (2010), cytoplasmic streaming does not distort the H_2_O_2_ signal too much, because cytoplasmic stream speeds are slow compared to diffusion over the short cellular distances that are relevant.

The maximal amount of H_2_O_2_ emitted in a pulse at the PM depends on the density of aquaporins able to transport H_2_O_2_ as well as on the flux per channel in the opened state. The density of aquaporins has been estimated to be 30 molecules per μm^2^ (Li et al., 2011) and the flux per channel is assumed to be 10^9^ molecules s^−1^ (Jensen and Mouritsen, 2006). The channel density in the PM is probably a parameter that the plant needs to be able to regulate in response to long-term changes in the water supply. The question is how many of the molecules passing through the aquaporins are H_2_O_2_ and how many are water. We assumed no selectivity, but there could be some, which would alter the maximal possible flux. We assumed an external concentration of 100 mM H_2_O_2_ and that could well be an overestimate. We also assumed that all the aquaporins in the PM can transport H_2_O_2_, which is probably not the case (Bienert et al., 2007; Maurel et al., 2009). Thus, it is possible that the maximum flux possible across the PM turns out to be a limiting factor for H_2_O_2_ signaling by diffusion.

Opening times for the synchronized channels down to 1 ms were considered and were required for emission of short pulses over a distance of 1 μm (Figure 3A). Since opening of aquaporins is regulated by reversible phosphorylation/dephosphorylation, this would require that the protein kinase and phosphatase involved can complete their work in considerably less than 1 ms. We do not know whether this is possible.

An alternative to signaling by short pulses is H_2_O_2_ signals that resemble harmonic oscillations, which allow for longer opening/closing times for the PM aquaporins that produce these signals. However, in order to produce an harmonic oscillation, it is required that channel closing starts just as the maximal opening state has been achieved, and that may be more difficult to imagine in terms of enzyme kinetics. In this connection, it is interesting that a theoretical study has shown that aquaporin opening may also be voltage gated (Hub et al., 2010). Furthermore, while harmonically oscillating signals are able to create higher signal amplitudes since the channels stay open longer than for short pulse signaling (Figure 11) they have longer refraction times and only allow for signaling at much lower frequencies (Figures 11 and 12B).

The concentration of H_2_O_2_ in plant tissues has been reported to be in the micromolar to low millimolar range and usually higher under stress conditions, assuming an even distribution in all cells and cell parts including the vacuole (Halliwell and Gutteridge, 2007; Møller et al., 2007; and references therein). However, the observation that the ROS probe CM-H_2_DCFDA [(5-(and-6) chloromethyl-2’,7’ dichlorodihydrofluorescein diacetate] does not give a signal in the vacuole (Bienert et al., 2007) may indicate that the vacuole contains little H_2_O_2_, which in turn indicates that the concentration in the rest of the cell, where signaling occurs, is even higher. We assume that the vast majority of this H_2_O_2_ is free (not bound), i.e., we assume the opposite of what is the case for Ca^2+^ for which the concentration of free cytosolic ions is three orders of magnitude lower than the total tissue content, due mainly to binding and sequestering inside organelles (e.g., Jafri and Keizer, 1995).

If H_2_O_2_ signaling via diffusion with resting levels below 1 μM is a general phenomenon, the waves would indeed have to contain very high peak concentrations in order to give a tissue average hundreds or thousands of times above the resting level. As we have demonstrated, that is very likely not the case, unless relaying stations exist. At higher resting levels in the cytosol for H_2_O_2_, the flux across the PM would become limiting and so would enzymatic degradation. In other words, the relatively high average levels of H_2_O_2_ observed to be present in plant tissues makes it highly improbable that information is transmitted via diffusing H_2_O_2_, unless relaying stations exist in the cytoplasm.

The tissue concentrations of H_2_O_2_ referred to above are, of course, averages and probably differ considerably between cell types. If we take a C3 leaf in the light, the photosynthetically active cells, such as the palisade cells, would have a massive turnover of ROS in the chloroplasts and in the peroxisomes as a result of photorespiration (Foyer and Noctor, 2003) and probably a relatively high cytosolic ROS concentration. In contrast, the leaf epidermal cells, which in many species contain no chloroplasts, would be expected to have a ROS concentration much lower than the average for the leaf. The epidermal cells are also often the first cells to confront invading pathogens, where ROS production is an early response, so it may be significant that it is also in these cells that the transmission of information from the PM by ROS diffusion would be more likely to take place because of a lower background concentration of H_2_O_2_.

It is a common observation that the cellular ROS production increases in plants in response to stress (Apel and Hirt, 2004). In non-photosynthetic cells, the presence of the mitochondrial alternative oxidase in the mitochondria is very important for keeping the cellular ROS level low, probably because it lowers the reduction level of the electron transport components that are responsible for mitochondrial ROS production (Maxwell et al., 1999; Møller, 2001). Consistent with this, the alternative oxidase is often induced by stress treatments (Gadjev et al., 2006) presumably to prevent the rise in ROS levels. It follows that ROS signaling is likely to occur in unstressed non-photosynthetic cells.

Relaying stations, such as the endoplasmic reticulum and the vacuole (Berridge, 1993; McAinsh and Hetherington, 1998; Stael et al., 2012), make up an important component in Ca^2+^signaling, but none has been identified for hydrogen peroxide in plant cells. In animal cells, mitochondria are possible relaying stations (Zhou et al., 2010) for superoxide signaling, which may or may not work for plant mitochondria, given that the properties essential for the mechanism are not well described for plant mitochondria (Møller, 2001; Reape and McCabe, 2008).

In plant cells, mitochondria, peroxisomes, and plastids are potential relaying stations for H_2_O_2_ signaling, as they are known to produce ROS (Møller, 2001; Foyer and Noctor, 2003; Apel and Hirt, 2004). In fact, recently it was observed that the membrane potential of individual mitochondria oscillates *in vivo* as well as *in vitro* with pulse intervals of 5–50 s (i.e., frequencies of 0.02–0.2 Hz) and that this oscillation was affected by the redox state of the mitochondria (Schwarzländer et al., 2012). This could be an indication of a signaling network possibly involving ROS.

Even if H_2_O_2_ signaling by frequency- and amplitude-modulated waves occurs, we have little idea of how these signals are decoded. H_2_O_2_ is able to regulate genes as demonstrated in bacteria. A general trend in this regulation is that it involves the oxidation of a transcription factor thereby affecting its DNA-binding affinity (Lee and Helmann, 2006; Imlay, 2008). Whether such a system can decode a frequency- or amplitude-modulated signal is not known.

## Conflict of Interest Statement

The authors declare that the research was conducted in the absence of any commercial or financial relationships that could be construed as a potential conflict of interest.
